# A roaring trade? The legal trade in *Panthera leo* bones from Africa to East-Southeast Asia

**DOI:** 10.1371/journal.pone.0185996

**Published:** 2017-10-24

**Authors:** Vivienne L. Williams, Andrew J. Loveridge, David J. Newton, David W. Macdonald

**Affiliations:** 1 School of Animal, Plant & Environmental Sciences; University of the Witwatersrand, Wits, South Africa; 2 Wildlife Conservation Research Unit, Department of Zoology, University of Oxford, The Recananti-Kaplan Centre, Tubney House, Tubney, Oxon, United Kingdom; 3 TRAFFIC East/Southern Africa, c/o IUCN ESARO, Hatfield, Pretoria, South Africa; National Zoological Park, UNITED STATES

## Abstract

The African lion is the only big cat listed on CITES Appendix II, and the only one for which international commercial trade is legal under CITES. The trade in lion body parts, and especially the contentious trade in bones from South Africa to Asia, has raised concerns spanning continents and cultures. Debates were amplified at the 2016 CITES Conference of the Parties (CoP17) when a proposal to up-list lions to Appendix I was not supported and a compromise to keep them on Appendix II, with a bone trade quota for South Africa, was reached instead. CoP17 underscored a need for further information on the lion bone trade and the consequences for lions across the continent. Legal international trade in bones to Asia, allegedly to supply the substitute ‘tiger bone’ market, began in South Africa in February 2008 when the first CITES permits were issued. It was initially unclear the degree to which bones were sourced from captive-origin lions, and whether trade was a threat to wild lion populations. Our original assessment of the legal CITES-permitted lion bone trade from South Africa to East-Southeast Asia was for the period 2008–2011 (published 2015). In this paper, we consolidate new information that has become available for 2012–2016, including CITES reports from other African countries, and data on actual exports for three years to 2016 supplied by a freight forwarding company. Thus, we update the figures on the legal trade in lion bones from Africa to East-Southeast Asia in the period 2008–2016. We also contextualise the basis for global concerns by reviewing the history of the trade and its relation to tigers, poaching and wildlife trafficking. CITES permits issued to export bones escalated from ±314y^-1^ skeletons from 2008–2011, to ±1312y^-1^ skeletons from 2013–2015. South Africa was the only legal exporter of bones to Asia until 2013 when Namibia issued permits to export skeletons to Vietnam. While CITES permits to export ±5363 skeletons from Africa to Asia from 2008–2015 were issued (99.1% from South Africa; 0.7% from Namibia) (51% for Laos), actual exports were less than stated on the permits. However, information on actual exports from 2014–2016 indicated that >3400 skeletons were exported in that period. In total, >6000 skeletons weighing no less than 70 tonnes have been shipped to East-Southeast Asia since 2008. Since few wild lions are hunted and poached within South African protected areas, skeletons for the legal trade appear to be derived from captive bred lions. However, confirmation of a 116kg shipment from Uganda to Laos, and reports of lion poaching in neighbouring countries, indicate that urgent proactive monitoring and evaluation of the legal and illegal trade is necessary in African lion range states where vulnerable wild lion populations are likely to be adversely affected.

## Introduction

*“Anger over lion bones sales”* was the first South African media headline to proclaim publicly the existence of a legal trade in African lion bones, allegedly to supply the substitute ‘tiger bone’ market in East-Southeast Asia (E-SEA) [1]. The December 2009 story provoked widespread outrage when it was revealed that a CITES permit had been issued to a farmer to legally sell lion bones. Unbeknownst to the public however, and contrary to what was reported, South Africa had issued its first CITES export permit 22 months earlier in February 2008 for ‘10 skulls/skins’ and ‘20 floating bones’ to an importer in Lao People’s Democratic Republic (Laos) (erroneously recorded on the permit as Vietnam) [2]. A second CITES permit was issued in July 2008 to export 35 lion skeletons and 16 skins, followed by a third permit four months later for 15 skeletons. But by the time evidence for the legal trade became public knowledge in December 2009, permits to export another 197 lion skeletons of captive-origin to Laos had already been issued.

**Fig 1:**
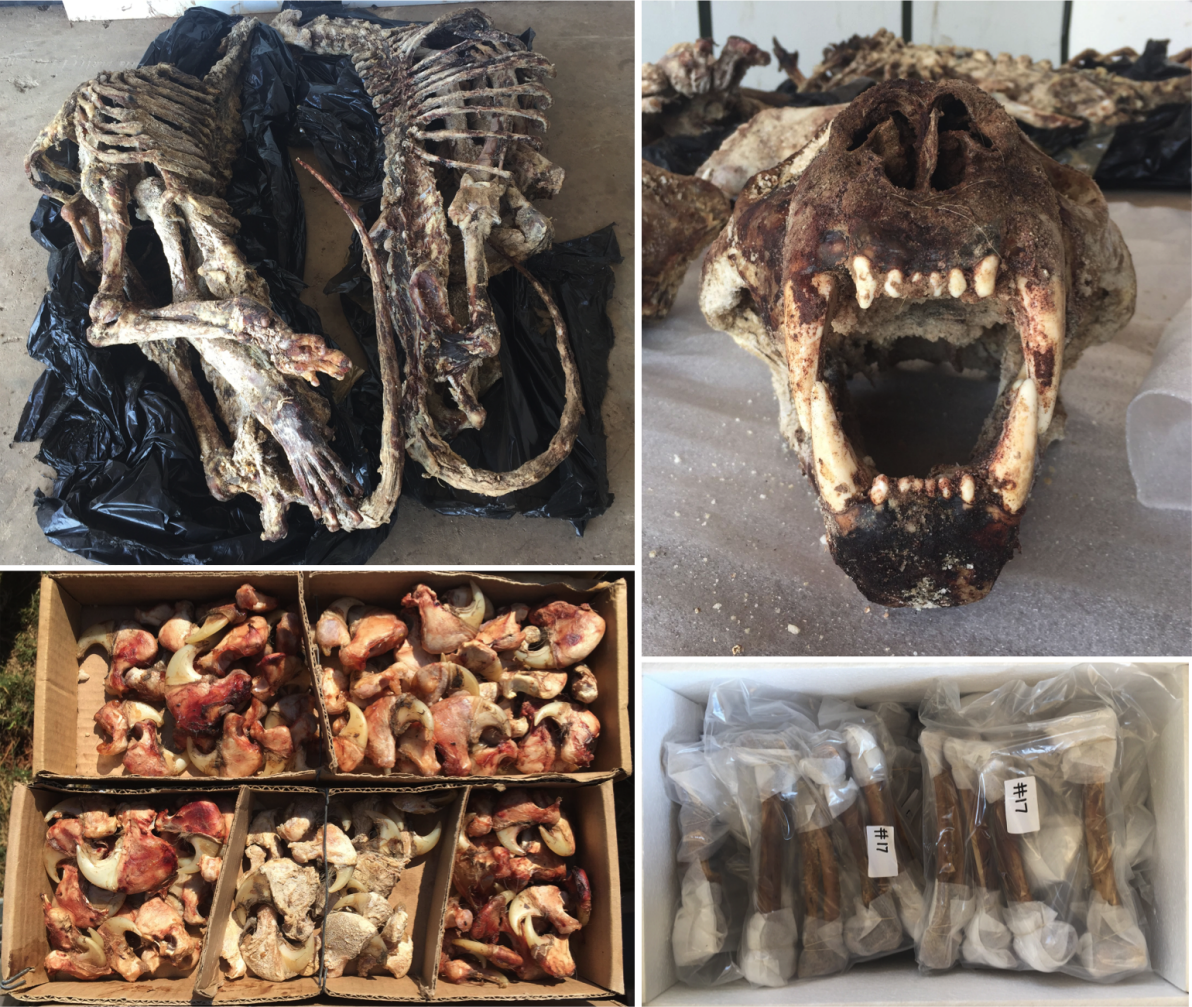
Lion skeletons, skull and claws before being sent for taxidermy, and a box of cleaned and prepared lion bones ready for export to Southeast Asia (bottom right) (V.L. Williams).

The existence of a market for lion skeletons in Southeast Asia, and the export of captive-origin bones from South Africa in 2008 (Fig 1), took the conservation community by surprise. The captive lion and canned hunting industries had previously courted controversy in 2007 when the South African Predator Breeders Association (SAPBA, now SAPA) challenged the Minister of Environmental Affairs over proposed regulations that captive lions could be hunted only after a 24-month ‘self-sustaining’ release period [3–5]–a case that SAPBA eventually won, and a matter that was later relevant to the lion bone trade. Two years later, when related concerns about the sources of skeletons for the lion bone trade emerged, pertinent questions raised included: were captive-bred and/or trophy hunted (including ‘canned-hunted’) lions the source of the bones, and was commercial and domestic trade an incipient latent risk that that could adversely affect vulnerable wild lion populations across Africa?

To answer these questions we undertook two investigations of the bone trade, starting in 2013. The first investigation was an assessment of the South African lion bone trade for the period 2008–2011 [2,6,7], and the second a pan-African stakeholder survey in 2014–2015 to record the then prevailing knowledge on the utilisation and trade of lion body parts (including bones) across current and former range states [8]. In the first investigation we concluded that skeletons from South Africa were typically a so-called ‘by-product’ of the trophy hunting industry that originated from captive-produced lions; and, despite there being no cultural history of consuming lion parts in Asia, the emergence and persistence of the South African–Asian lion bone trade is inextricably linked to the market for tiger products (and other large felids) [2]. However, while the trade did not appear to be impacting specifically on South African wild lion populations in formally proclaimed protected areas, the status and collateral impacts of the bone trade on wild lion populations in other range states was subject to debate and concern and was largely undocumented [2]. The second investigation revealed that domestic utilisation and international trade of lion bones within and among African countries for traditional purposes (especially zootherapeutic, or medicinal) is an evident cultural stimulus for trade in addition to the demand from markets in East-Southeast Asia [8]. Hence, there are concerns that the legalised sale of lion bones, supplied by captive-bred lions, from South Africa is (i) impeding efforts to curb the tiger trade because access to lion parts might be perpetuating and/or expanding the market for large felid bones, and thereby rekindling efforts to poach tigers as the demand is supplied, and (ii) abetting the illegal acquisition and trade in lion bones and derivatives across Africa (seemingly evinced through the rise in incidences of lion poaching and trafficking).

The debate on the international trade in lion derivatives came to the fore at the October 2016 17^th^ Conference of the Parties to CITES (CoP17). Nine lion range states proposed that *Panthera leo* be up-listed to Appendix I status based on threats to the species, including the international bone trade [9]. However, the proposal was not strongly supported because *“neither the biological nor the trade criteria required to list the African Lion under Appendix I were met”* [10]. A controversial compromise was reached, allowing African lion to remain on Appendix II but with the following annotations: (i) a zero annual export quota for specimens of bones, bone pieces, bone products, claws, skeletons, skulls and teeth removed from lions in the wild and traded for commercial purposes, and (ii) South Africa must establish a national export quota for commercial trade in lion bones, bone pieces, bone products, claws, skeletons, skulls and teeth derived from captive breeding operations [10,11]. Furthermore, studies on the legal and illegal trade in lions (including the bone trade) must be conducted, in part to inform the quota setting process, and so that CITES Parties can re-evaluate the status of lions at CoP18 in 2019. The African lion is the only big cat listed on CITES Appendix II and as such, the only one for which international commercial trade is legal under CITES, up until the CoP17 Decision, when it was limited to a quota for South African exports. The 2017 lion bone export quota was set at 800 skeletons, with or without the skull, in July 2017; however, no export quota has been set for teeth, claws or individual bones.

Considering the requirement for information on the lion bone trade, the purpose of this paper is to re-examine information for the East-Southeast Asian lion bone trade from 2008 by (i) consolidating and updating new information available for the period 2012–2016, and (ii) including information on concurrent legal trade reported from other African countries. Hence this paper reports on the export of lion bones from Africa to East-Southeast Asia from 2008–2016. Furthermore, we contextualise the basis for global concerns regarding the lion bone trade by reviewing the history of the East-Southeast Asian trade and its relation to tigers, poaching, wildlife trafficking and Asian wildlife trade syndicates.

## Rise of the lion bone trade to East-Southeast Asia

The illegal trade in tiger body parts is a persistent and significant threat to wild tiger populations across Asia [12,13,14], and their bones (an ingredient in Traditional Chinese Medicines, TCM) are one of the most lucrative products sold on the illegal wildlife market [15]. But while there are diverse strategies, policy interventions and efforts that are intended to protect tigers by securing habitats and landscapes, prevent poaching, strengthen compliance with existing trade bans, and effect constructive trade reduction measures, felid substitutes (most notably lions, but also leopards) for tigers are maintaining a supply of bones and may be foiling efforts to curtail the market [2].

As Chinese wild tiger populations rapidly declined, it was noted that purveyors of tiger products turned to other tiger range-states, and later other large Asian felids, to source bones. Tiger poaching in India, for example, was noted in the mid-1980s [16–19] and eventually incidents involving leopards and the Gir/Asiatic Lion (*Panthera leo* subsp. *persica*) were reported there about 20 years later [19–22]. In 1997, Khoshoo predicted that derivatives from big cats would eventually be substituted for tiger parts in Chinese medicines: *“once tiger is decimated*, *the next target will be lion*, *followed by leopard (even bear) and all other felines from Asia and Africa”* [23].

An increased prevalence in tiger bone substitutes was observed across East Asia and in shops selling Traditional Asian Medicines (and associated purported tonic preparations) in the USA and Australia from the mid-1990s [14,24–27]. Images of lions appeared on labels of manufactured Chinese medicines c.1995 [24,27], but there was no evidence then that bones from wild lions were being used–however, there was speculation that bones from captive animals in China were being substituted illegally [27]. Sales of products with lion bone are not banned in China [14], hence using substitutes (or, pretending to use *Panthera* species substitutes by mislabelling the products and/or excluding derivatives from the mixtures) was a way to avoid attracting regulatory action for products that did not contain tiger [S. Broad, pers. comm., March 2015].

An investigation by TRAFFIC in 2005 confirmed that African lions were an ingredient in ‘tiger’ ‘bone strengthening wine’ [28]. A company in southeast China was given permission to produce the wine, but ‘Panthera leo’ bones were listed as the approved ingredient [12,27,28]. Furthermore, a nearby tiger breeding farm that was a subsidiary of the company also kept African lions on its premises [22,29]. Despite the company insisting that the product was made from tiger carcasses, an investigation by the Chinese government in 2006 reported that *“only 16 legally obtained lion carcasses were found*, *and no tiger bones were used to produce the wine”* [28].

Khoshoo’s [23] projection that lion would be a target was confirmed a decade later. In March 2007 the first of three incidents occurred that police said implicated TCM as the motive behind the poaching of at least eight Asiatic lions over a six-week period in the Gir National Park, Gujerat, India, which led to a gang of poachers being convicted in 2008 [2,19,21,22,30–33; Wildlife Protection Society of India, pers. comm. 11/05/2014]. These incidents caught Indian officials unawares since no one suspected that the market for medicines would be implicated in, and lead to, incidents of lion poaching [19].

Preceding and coinciding with these incidents, and ones that followed in South Africa in 2008, were the series of policy interventions (at national and international levels [13]), including Decisions and Resolutions adopted at various Conferences of the Parties to CITES pertaining to Asian Big Cats (Tiger, Leopard, Clouded Leopard, Snow Leopard) that were intended to diminish and ban the trade in tiger and Asian felid products, enhance enforcement and compliance, and protect tigers and other Asian big cats. In response, syndicates involved in the illicit trade found legislative loopholes and alternative sources for tiger bones to circumvent (and/or ignore) trade bans and other measures that delimited their activities [2]. Henry [26] reported that *“as tigers received greater protection and attention*, *the demand for tiger parts used in traditional medicines shifted to other Asian big cats and further threatened their survival”*. A Decision taken at CITES CoP14 in June 2007 might also have been an inadvertent factor in the beginnings of the South African lion bone trade, *viz*. *Decision 14*.*69*. The Decision stated that countries with tiger breeding programmes (such as China, Vietnam, Thailand, Laos) should phase out breeding for commercial purposes and limit the size of their captive populations [34–37] (note: there is no indication that captive tiger populations have been reduced in response to this Decision, which thus led to a series of new decisions at CoP17 focussed on tiger farming [38]). Eight months later in February 2008, the first CITES permit to export lion bones from South Africa was issued–but warnings of Asian interests in African lions, and potential threats to another of the continent’s big five flagship species, were only sounded the following year.

In March 2009, conservation officials were alerted to an illicit trade in lion parts when Nguyen Van Hai, a Vietnamese immigrant, was arrested in Pretoria (South Africa) for being in possession of several lion carcasses [2,39–46]. Detectives found *“recently-executed lions and…rhino”* on the premises, and speculated that he was involved in killing endangered African mammals ‘to-order’ for a syndicate operating from the ‘Far East’ [41,45]. Thereafter, there was no overt evidence of trade for the next eight months until December 2009 when it was reported that there was a growing trade in bones to Asia (sourced from hunting facilities) after a lion farmer had been issued a CITES permit to legally sell bones [1,2,47]. To Asian importers, skeletons from captive-origin lions were plentiful, cheaper, and mostly legal alternatives to tigers and other large felids. And, since it was known: (i) that demand was driven from East-Southeast Asia, (ii) that big cat bone traders accepted lion bones as an alternative to tiger bone, and (iii) that lion bones were reportedly being passed off as tiger bones in medicines and tonics, the conservation world was becoming increasingly nervous about the trajectory of the trade and what impact it might have on wild lion populations.

The next arrests occurred in June 2011 when two Thai men (Phichet Thonghpai and Punpitak Chunchom) were found with lion bones in Johannesburg [48,49]. They admitted in court that they worked for *“Vichai Company”* (the Xaysavang Export-Import Company, headed by Vixay Keosavang, in Laos [22,47,50]), and that the main business of the company was to trade in lion bones [48,51]. Furthermore, they said they *“were sent to South Africa by the company to view and approve lion bones to be bought and shipped to the company”* [48,51]. Thonghpai also admitted that the *“company for which I worked is usually contacted by farm owners in South Africa and advised that they have lion bones for sale”* [48]. Both men were fined and repatriated [48]. A month later in July 2011, police arrested the leader of a rhino poaching syndicate––Chumlong Lemtongthai–at the same residence as Thonghpai and Chunchom [47,49,52,53]. Hence, Lemtongthai was part of the same syndicate working for Xaysavang and also trading in bones [[47,52,54,55]. Details of Lemtongthai’s rhino poaching activities are covered extensively by Rademeyer [47] and TRAFFIC [56]. Chunchom, however, was rearrested on arrival in Johannesburg in November 2011 [57]; it was suspected that he had returned to buy lion bones and ‘engage in illicit activities’–but this time he was also accused of running a rhino horn smuggling syndicate with Lemtongthai [51,58,59]. Chunchom’s case was joined with Lemtongthai’s and the case came to trial a year later; while Lemtongthai was eventually sentenced in November 2012 to 40 years imprisonment for charges relating to illegal rhino hunting and horn exports, the charges against Chunchom were dropped and he fled the country illegally [2,60].

Lemtongthai appealed his sentence twice [61,62]; admissions published in court documents after his appeal in 2014 (when his sentence was reduced to 13 years) publically revealed: (i) that the Xaysavang Company dealt in the trade of rhino horn, lion bones, teeth and claws; (ii) Lemtongthai was sent to South Africa by Keosavang to enquire about the purchase of lion bones; (iii) on Lemtongthai’s arrival in South Africa, he saw various adverts for the hunting of the big five, including rhino; and (iv) Keosavang said he would fund any trade in rhino horn [62]. It was also reported that he wanted to buy 300 sets of lion bones [63,64]. Thus, the admissions made by Lemtongthai, Chunchom and Thongphai during their 2011–2014 court cases, and other evidence provided by informants to our research, suggests that Xaysavang’s involvement in the lion bone trade precedes their involvement in the rhino horn trade, and that the company started legally procuring lion bones from farmers c. February 2008 when the first CITES export permit was issued [2]. However, there are allegations that the illegal export of bones and Xaysavang’s relationship with South African lion breeders through Lemtongthai commenced earlier [e.g. 54]. Furthermore, it seems likely that the surge in the number of rhinos killed in poaching incidents from 2008 was entangled with Xaysavang and the commencement of the legal lion bone trade the same year [2].

Keosavang, however, is reportedly no longer a kingpin in the Laotian wildlife trade, having allegedly stepped back in 2014 due to pressure placed on him by the US government, and also the revoking of his licence to trade wildlife by the Lao government in January 2014 [50,54,65]. His step down coincides with the 2014 decline of bone exports to Laos (see Table 1). But, there are other Laotian-, Vietnamese- and Thai-based customers and syndicates involved in the lion bone trade (see [54,65]), and the legal trade will continue from South Africa while a quota is in effect. The extent to which the trade is conducted through lawful sources is examined next.

**Table 1:** Total number of lion skeletons and bodies ^a^ originating from Africa listed on issued CITES permits and destined for East-Southeast Asia from 2008–2015. Figures do not represent the actual annual exported quantities ^b^.

Year	Laos	Vietnam	Thailand	China	Total
*Skeletons (SKE)*					
**2008**	60	0	0	0	60
**2009**	171	2	0	48	221
**2010**	240	117	0	0	357
**2011**	531	64	20	2	617
**2012**	87	85	0	0	172
**2013**	974	309	14	0	1297
**2014**	433	892	0	0	1325
**2015**	230	936	148	0	1314
**Total SKE**	2726	2405	182	50	5363 ^c^
*Bodies (BOD)*					
**2008**	0	0		1	1
**2009**	0	0		0	0
**2010**	0	0		10	10
**2011**	0	0		21	21
**2012**	61	104		15	180
**2013**	0	0		27	27
**2014**	0	2		0	2
**2015**	0	0		0	0
**Total BOD**	61	106		74	241 ^d^
***Total no*. *SKE & BOD***	*2787*	*2511*	*182*	*124*	*5604* ^b^
***Total %***	*50%*	*45%*	*3%*	*2%*	

^a^ The CITES database lists more bodies, but most records were converted to skeletons (see Methods)

^b^ The actual annual exported quantities are less than what was listed on the issued CITES permits because traders do not typically export all of what they applied to export, and/or they don’t use the permit in the same year it was issued

^c^ 99.1% (5316 SKE) from South Africa; 0.9% (47 SKE) from Namibia (all to Vietnam 2013–2015) (South African provincial data in Table 2)

^d^ 97.9% (236 BOD) from South Africa; 2.1% (5 BOD) from other African countries viz. Namibia (2 BOD), Tanzania (2 BOD) and Zimbabwe (1 BOD) (all to China) (South African provincial data in S1 Table)

## Methods

### Information sources

Trade data on legal exports of lion bones from Africa were obtained from: (i) the online CITES Trade Database maintained by UNEP-WCMC (https://trade.cites.org/) for 2008–2015; (ii) unpublished data supplied on request by the South African Department of Environmental Affairs (DEA) and the South African CITES Scientific Authority, including the annual reports of issued permits that were submitted to the CITES Secretariat for 2008–2015; and (iii) actual export data for 2014–2016 provided by the freight forwarding company that has handled most of the bone consignments destined for East-Southeast Asia (E-SEA) since 2013. The export data from the freight forwarding company were only supplied with the consent of their customers (i.e. six of the main traders of lion bones in South Africa, who buy bones from farms and hunting facilities) and on the strict condition that their identities were not revealed; only the corresponding author of this paper has communicated with the people concerned. This company and the traders also supplied pertinent anecdotal information that are included as anonymous personal communications. Since evidence showed that the legal lion bone trade to E-SEA commenced in early 2008, and that there were only sporadic, low-volume records of lion products being exported there up to 2007 (see Figure 24 in [2]), we have only included permit reports from 2008 onwards.

CITES export permit data indicate the total quantity (e.g. sets of bones) that specific export permits were issued for; hence, an exported consignment should not exceed the quantity stated on the permit. Actual quantities of legally exported bones can only be deduced from (1) records of CITES permits that have been inspected and ‘endorsed’ by a nature conservation inspector at the port of exit (for which we had access to an incomplete set of records while preparing this manuscript) (see Results), and/or (2) from the air waybills (AWB) generated by freight forwarding companies [2], and/or (3) from records kept by the exporting traders. Data supplied by the freight forwarding company from the AWBs from 2014–2016 was on behalf of the lion bone traders, and these data contained: (i) combined monthly totals of the sets of bones exported, and (ii) the destination countries (but not the names of the cargo recipients) in E-SEA.

### Cross-checking and correcting the CITES trade data

The annual CITES reports are the most accessible means available for monitoring the maximum levels of legal international trade [66], but they rely on consistent and accurate reporting by CITES Parties. We had concerns over the fidelity of the reports submitted to the Secretariat when cross-checking the detailed South African annual reports with the CITES Database (compared by using the variables: importer, reported quantities, trade term, purpose and source). One concern relates to the interpretation of trade terms–for example, the CITES trade term ‘bone’ (BON) is different to ‘skeleton’ (SKE). Whereas ‘bone’ is in units of individual bones (e.g. floating bones), and/or the mass thereof, ‘skeleton’ refers to the number of *“substantially whole skeletons”* [66]. A ‘skeleton’ is further differentiated from a ‘body’ (BOD), which refers to *“substantially whole dead animals*, *including…whole stuffed hunting trophies*, *etc*.*”*. During crosschecking, we found that BON, SKE and BOD destined for E-SEA were inconsistently classified on South Africa’s annual reports–in part because (1) there is no CITES trade term guideline for interpreting the description ‘carcass’, and (2) there were different interpretations of what constitutes a ‘set’ of lion bones. For example: (i) *5 ‘scull’ & bones of 5 carcasses* were captured as ‘*5 SKE*’; (ii) *32 carcasses* were captured as ‘*32 BON*’ (instead of 32 SKE); (iii) *50 carcasses (947kg)* were captured as ‘*50 BOD*’ (instead of 50 SKE); (iv) *117X9 Bones* were captured as ‘117 BON’ (instead of either 1053 BON or 9 SKE, partially complete, see below), (v) *bones of 15 lions* was recorded as ‘*15 BON*’ (instead of 15 SKE), and (vi) *2 (two) sets of bones*, was recorded as ‘*2 BON*’ (instead of 2 SKE). These inconsistencies were consequently incorporated into the Trade Database and thus some of the quantities available for BON, SKE and BOD for South Africa are misleading, sometimes inflated, and punctuated with errors. [*Note*: under the 2017 quota system, record anomalies should not occur because (i) only complete skeletons are allowed to be exported, with or without the skull, and (ii) issued CITES export permits will show the actual quantity of skeletons permitted in a shipment per trader (viz. number, and the total weight), instead of quantities that traders used to sometimes ‘guestimate’ they could export when they applied for permits from Issuing Authorities prior to procuring bones from facilities].

These inconsistencies thus necessitated that all the lion bone data on the CITES Trade Database be crosschecked with the original DEA information to standardize, correct and/or reclassify records wherever appropriate (as done in the examples described). Further examination of the permits established that ‘bones’ exported for the lion bone trade to E-SEA are usually ‘sets’ of lion bones and thus ‘skeletons’ of varying degrees of completeness (where one ‘set’ comprises bones derived from the skeleton of one lion). Bones like the skull, jaw and clavicles (paired ‘floating bones’) are typically absent from a set if the lion was a hunting trophy. From our estimates for the period 2006–2011, ±14% of skeletons of trophy-hunting origin were complete sets [2] (a lion bone trader subsequently confirmed that it was unlikely that >20% were complete sets [Anonymous, pers. comm., July 2017]). Hence, while lions have up to 309 bones (including teeth and sesamoids), a set from a trophy could have up to 206 bones (minus the skull, jaw, clavicles, teeth and sesamoids), or a partial skeleton could have a set of 117 bones if the vertebrae are excluded [2].

Wherever the mass of a set of bones was captured on a CITES permit, it was converted to units of skeletons (e.g. 1573 individual bones declared to weigh 107.5kg was calculated to be equivalent to ±11 SKE following Williams et al [6]). It was by these means that some anomalous permit declarations were identified and corrected for, such as 947kg of bones being listed as 50 SKE instead of ±100 SKE (see Williams et al [6]). The biggest error detected was for ‘2910 SKE’ and ‘14 SKE’ destined for Thailand in 2013; on re-examination of the original provincial record, the permit entry was for *‘2910 bones from 14 skeletons’*–hence this record was corrected in our analyses to reflect 14 SKE only. In re-interpreting the data, all but one of the records for ‘bones’ listed on the Trade Database could be subsumed within the category ‘skeletons’. A 2013 permit for ‘531 BON’ destined for Vietnam was not converted to SKE because there was no corresponding information in the annual report from DEA (compiled for CITES) to compare with.

Since the issued CITES permits are listed provincially in the annual reports submitted by DEA to the CITES Secretariat, we could request that anomalous permit records be re-examined by the provinces. Consequently, an error was found in the ‘country of import’ on the first permit issued by the Free State province (South Africa) to export lion bones in February 2008. Despite ‘Vientiane, Lao PDR’ being typed on the original permit, the country was incorrectly listed as VN (ISO code for Vietnam) instead of LA (ISO code for Lao PDR). Further queries for the other permits issued that year also established that all the 2008 records on the CITES Trade Database for lion ‘bodies’ exported to ‘VN’ were incorrect, and they were amended to ‘skeletons’ exported to ‘LA’ accordingly.

Since we could not satisfactorily quantify the extent of illegal trade for lion bones, this aspect is not mentioned much in this paper. TRAFFIC International, which annually publishes records of some seizures and prosecutions that have come to its attention, had only published two cases to date of seizures involving African lion bones and skeletons in E-SEA (which incidentally have not been reported on the CITES database as source code ‘I’), and three cases involving Asian nationals arrested in Africa with claws and/or teeth [67–69]. Furthermore, there are no CITES records of (i) legal inter-Asian trade in lion bones, medicines or derivatives, or (ii) illegal trade (i.e. seizures) between Africa and E-SEA for lion products (using source code I). However, the US has made four seizures since 2009 of lion derivatives/medicines from China, including one coded ‘commercial’ for 200 units. This suggests commercial ‘medicinal’ trade in lions is not restricted to Asia and Africa; like the tiger trade, it may involve the wider Asian diaspora [K. Nowell, pers. comm., May 2017].

Throughout the paper ‘East-Southeast Asia’ (E-SEA) collectively refers to key destination countries for lion bone exports, namely China, Laos, Thailand and Vietnam. The E-SEA sub-region technically comprises 22 mainland and maritime countries sometimes referred to colloquially as the ‘Far East’. The 18 other countries or territories in E-SEA were excluded from the study because there were no records of legal lion bone trade in the 2008–2015 period. South Korea had, however, reported at least 29 lion bodies, of which three were allegedly wild-sourced (W) and originated in South Africa and the remainder were captive-bred (C) from Europe; the trade purpose was mainly listed by the importers as being for ‘circus or travelling exhibition’ (Q), whereas the exporters listed the purpose as commercial (T).

## Results and discussion

### The African lion bone trade: 2008–2016

In addition to hunting trophies, African countries have issued permits to legally export 22 other categories of lion body parts since 1977 (CITES Trade Database). Lion skeletons, bones and bodies have been exported to E-SEA since 1998, and especially since 2008. South Africa is the primary exporter (with bones mostly obtained from trophy hunted captive-bred lions), however other African countries have also issued CITES export permits (all wild-origin).

The CITES export data presented here for 2008–2015 are based on the adjusted quantities listed on the export permits issued for skeletons (SKE) and bodies (BOD) (see Methods)–in other words, quantities traders had usually ‘guestimated’ they could export when they applied for the permits, and *not* the actual quantities exported. However, most traders say they tend to use the entire permit, so actual exports should be close to the quantities listed on the issued permits [Anonymous, pers. comm., July 2017].

From the permit endorsement records it was noted that some exported consignments were smaller than the maximum allowed by the corresponding permit, and some permits were not used in the same year they were issued. Lion bone traders said that this happened quite frequently in the past because hunting establishments had a tendency to stockpile all, or most, of the skeletons resulting from hunts in a year until ca. November, after which they would sell them to “[lion bone traders] *to assist with travel expenses during January and February when most of the international* [hunting] *tradeshows take place*” [Anonymous, pers. comm., July 2017]. And, since the traders were unable to complete the applications for permits (including CITES) in time due to the December vacation period in South Africa, the export of those stockpiled bones was typically delayed until January/February of the following year [Anonymous, pers. comm., July 2017]. However, bone traders also said that uncertainty in the industry from January 2016 resulted in this practice (of stockpiling) being abandoned, and most hunting farms sold bones on a monthly basis for the rest of that year [Anonymous, pers. comm., July 2017].

As noted in the Methods, records of CITES export permits that are ‘endorsed’ at a port of exit can be used to compile the annual number of exported skeletons. To endorse a permit requires a nature conservation official to inspect the shipment, certify the quantity declared, and return the third page of the permit to the Issuing or Management Authority [2]. However, CITES permits for lion bone shipments were not consistently endorsed until April 2015; hence, we were unable to determine what proportion of skeletons/bodies listed on the permits were documented to have been exported prior to that period. Officials at OR Tambo International Airport (ORTA, Johannesburg) (reliably believed to be the only port of exit for South Africa’s legally exported lion bones) keep a record of endorsed permits and submit these to the South African Management Authority (DEA, pers. comm., 8 March 2017). Data supplied to us for permits endorsed at ORTA for the period October 8, 2015 to November 26, 2016 showed that 89% of the bone quantities listed on those permits issued in that period were exported. However, this data set is incomplete. Hence, except for the air waybill (AWB) data from the freight forwarding company on actual exports from 2014–2016, our results are a guide to the maximum quantities that could have been legally exported from Africa to E-SEA from 2008 to 2015. Traders say that under the 800 skeleton per year quota, 100% of the permit will be used because the maximum quantity allowed for 2017 is less than what they can be supplied with [Anonymous, pers. comm., July 2017].

#### Skeletons: CITES permit records

Quantities listed on CITES permits for the worldwide legal export of lion skeletons before 2008 totalled 14 specimens that were mostly wild-sourced (W) for scientific (S) and educational (E) purposes (average <1yr^-1^ from 1982–2007; six from Africa). The only export permits South Africa issued in that period was for three skeletons to Denmark in 2001. In 2008, South Africa issued the first permits to export 60 captive-origin (C) skeletons to Laos for ‘personal’ purposes (P) (erroneously reported on the CITES Trade Database as 60 ‘bones’ to Vietnam). Thereafter, the quantities reported on permits issued in Africa grew at a rapid rate and averaged 314yr^-1^ from 2008–2011, but 1312yr^-1^ from 2013–2015 (Table 1). In the period 2008–2015, permits allowing ±5363 skeletons to be exported from Africa to E-SEA were issued (5316 from South Africa; 47 from Namibia in 2013–2015). Laos was the primary destination (2726 SKE; 51%), followed by Vietnam (2405 SKE; 45%), Thailand (182 SKE, 3%) and China (50 SKE; 1%) (Table 1).

We have not established reasons for the 2012 drop in the number of SKE listed on permits (Table 1)–although the number of BOD that year was more than five times the average for 2011 and 2013 (Table 1). However, lion bone traders said that the quantities they exported in 2012 increased from 2011 and they therefore attribute the 2012 figure to errors in record keeping, and/or incorrect data capture, by the provincial CITES permit Issuing Authorities [Anonymous, pers. comm., April 2017].

**Fig 2:**
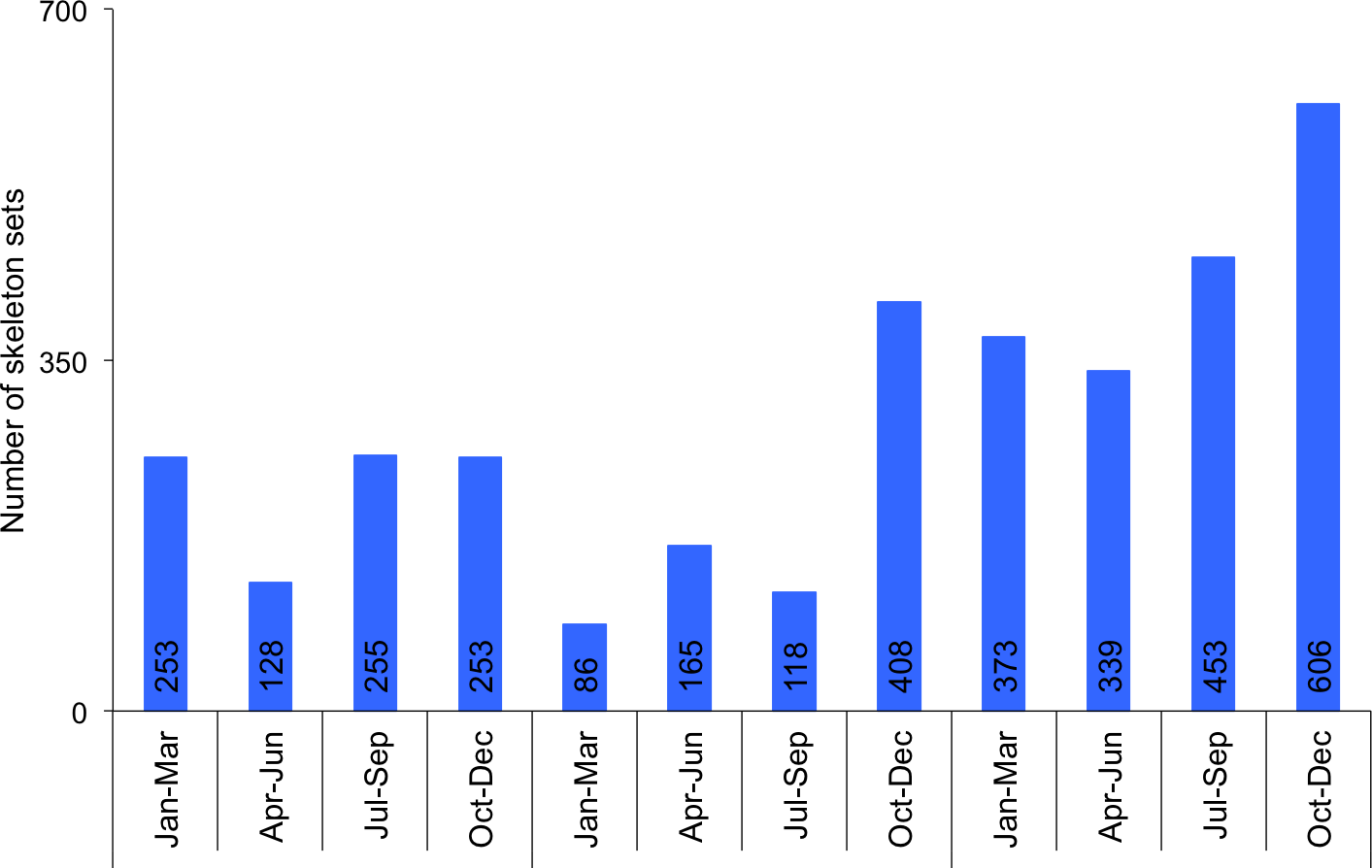
Actual annual quarterly exports of sets of lion skeletons from South Africa to East-Southeast Asia from 2014–2016, obtained from air waybill records provided by a freight forwarding company handling the exports on behalf of six lion bone traders.

**Fig 3:**
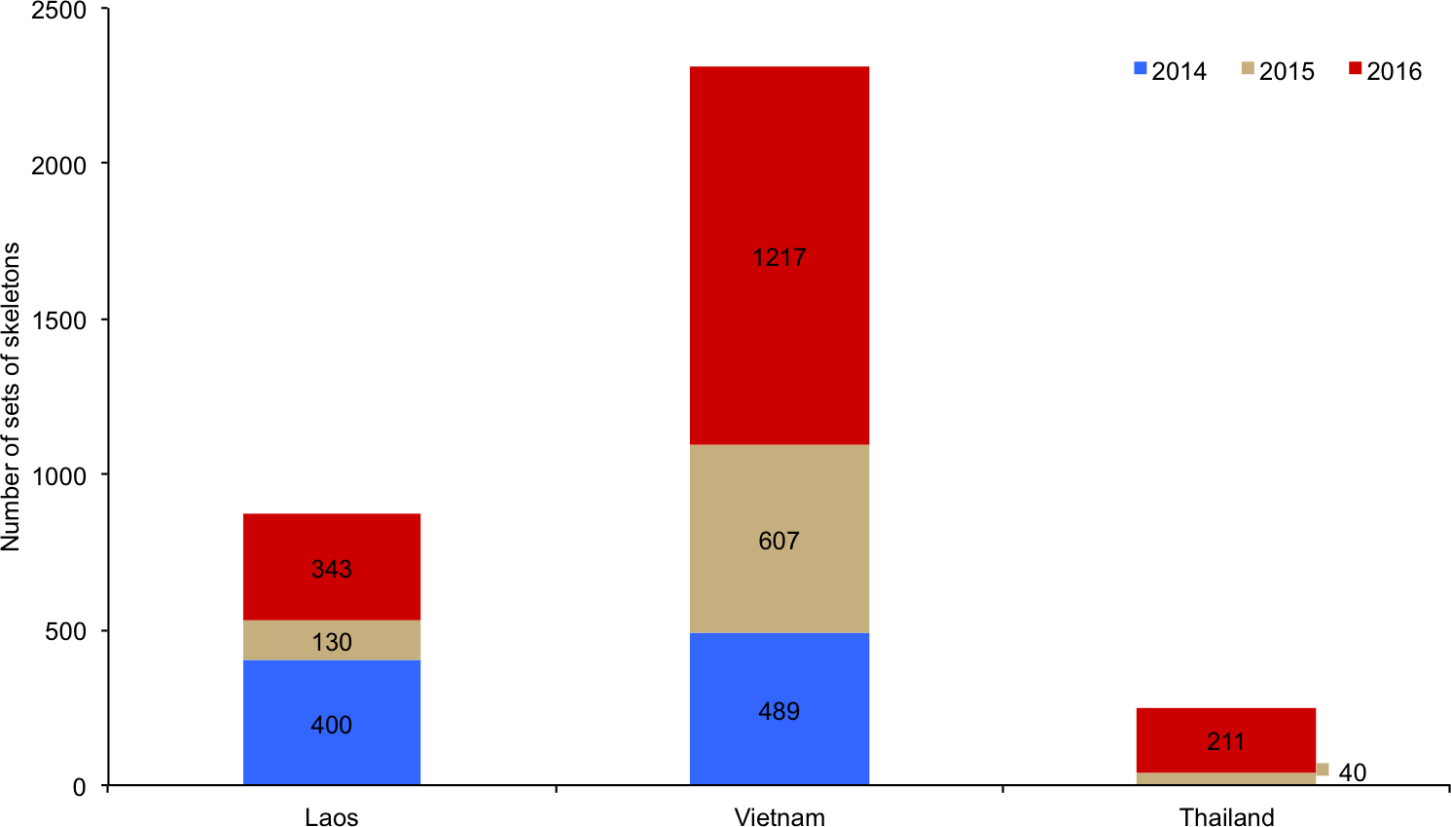
Actual annual exports of sets of lion skeletons from South Africa to Laos, Vietnam and Thailand from 2014–2016, obtained from air waybill records provided by a freight forwarding company handling the exports on behalf of six lion bone traders.

In 2014 and 2015 there was a sharp decline in the annual number and proportion of skeletons that bone traders applied to export to Laos (Table 1). The quantities dropped from 76% of SKE for 2008–2013, to 25% of SKE for 2014–2015. The drop was attributed to be a consequence of: (i) the Laos-based Xaysavang company, the primary importer of bones, being weakened by pressure from the USA and having its licence revoked to trade wildlife in January 2014 (see later), and (ii) a seven-month commercial trade suspension of CITES-listed species that Laos received from March 2015 because of their failure to submit a National Ivory Action Plan (NIAP) timeously and in accordance with recommendations previously adopted by the CITES Standing committee [70–72; Anonymous, pers. comm., April 2017]. After the NIAP was received, CITES Parties lifted the suspension of trade in September 2015 and bone exports to Laos resumed in November 2015 (Anonymous, pers. comm., April 2017). Importantly, these factors resulted in (i) a reduction in the average monthly exports of skeletons in 2015 [Anonymous, pers. comm., April 2017] (see also Fig 2), and (ii) trade being diverted to other countries because South African bone traders sought new customers in E-SEA. Trade was mostly diverted to Vietnam during the involuntary market restructuring (Table 1; Fig 3), however some traders ceased exporting for eight months in 2015 because their only customers were in Laos [Anonymous, pers. comm., April 2017]. Regarding their customers, South African traders suspect there are 3–5 main customers for lion bones in E-SEA–however, traders said that they typically do not deal with these customers directly and that they liaise instead with numerous agents representing these customers [Anonymous, pers. comm., April 2017].

Besides South Africa, only Namibia is known to have issued CITES export permits for skeletons up to 2015 (99.1% and 0.9% of the total quantity respectively). Furthermore, only South Africa and Namibia have issued permits to export skeletons to non-Asian destinations (three from South Africa to Australia in 2014; six from Namibia to an unknown destination in 2013). Hence South Africa has issued permits to export 99% of all lion skeletons listed on the CITES Trade Database up to the end of 2015. As noted earlier, our figures include the reclassified permit entries and will thus not align completely with the CITES database entries.

It remains to be seen whether other African countries issued CITES permits to export lion bones to E-SEA in 2016, but there are no records of non-African countries exporting skeletons there. However, there is AWB evidence for a 116 kg consignment of lion bones being exported from Uganda to Laos in 2016 (±10–12 SKE, using [6]) [V.L. Williams, pers. obs., May 2017]; whether this consignment was legal and had a CITES permit is unknown. Furthermore, several South African lion bone traders (i) believe there are illegal, but not substantial, exports of bones from other African countries, and (ii) heard rumours that Zimbabweans were investigating exporting skeletons, but they had no evidence that trade had actually occurred [Anonymous, pers. comm., April and July 2017]. The CoP17 annotation, however, will quash any plans by lion range states other than South Africa to legally export bones from 2017.

The main South African provinces issuing CITES permits from 2008–2015 were the Free State (2023 SKE; 38%), Gauteng (1371 SKE; 26%) and North West (1342 SKE; 25%) (Table 2). Except for Gauteng, these provinces are the main role-players in the lion hunting and/or captive-breeding industries. The Free State has the most lions in captivity, but most trophy hunting takes place in North West [2]. An audit of captive breeding facilities in South Africa in 2016 revealed that 29% of these facilities had sold lion bones in the past [DEA, pers. comm., January 2017]. While the Free State has consistently issued permits to exporters from the province since 2008, Gauteng exporters are only recorded from 2013. Lion bones, however, most likely do not originate from the same province in which a CITES permit is issued–especially in the case of Gauteng-origin permits. The traders buy skeletons from multiple facilities in different provinces, consolidate the shipments, and then apply for permits from a province (usually their home province) to export multiple sets of bones, irrespective of skeleton origin [2; Anonymous, pers. comm., July 2017]. In 2011, DEA published the names of six South African exporters and four or five E-SEA importers [73]; we presently know there to be six exporters (five from Gauteng, none of whom own a breeding or hunting facility), only one of whom was listed in the 2011 DEA document.

**Table 2:** Quantities listed on CITES permits issued by South African provinces to export lion skeletons (SKE) to East-Southeast Asia from 2008–2015.

Year	Free State	Gauteng	North West	Eastern Cape	Mpumalanga	Limpopo	Unknown	*Total* *SKE*
**2008**	60							*60*
**2009**	15				158		48	*221*
**2010**	83		221	53				*357*
**2011**	116		437	64				*617*
**2012**	68		25	77			2	*172*
**2013**	282	247	659	48			48	*1284*
**2014**	781	439				6	76	*1302*
**2015**	618	685						*1303*
***Total no*. *SKE***	*2023*	*1371*	*1342*	*242*	*158*	*6*	*174*	*5316*
***Total %***	*38%*	*26%*	*25%*	*5%*	*3%*	*0*.*1%*	*3%*	

#### Skeletons: Air waybill records

**Fig 4:**
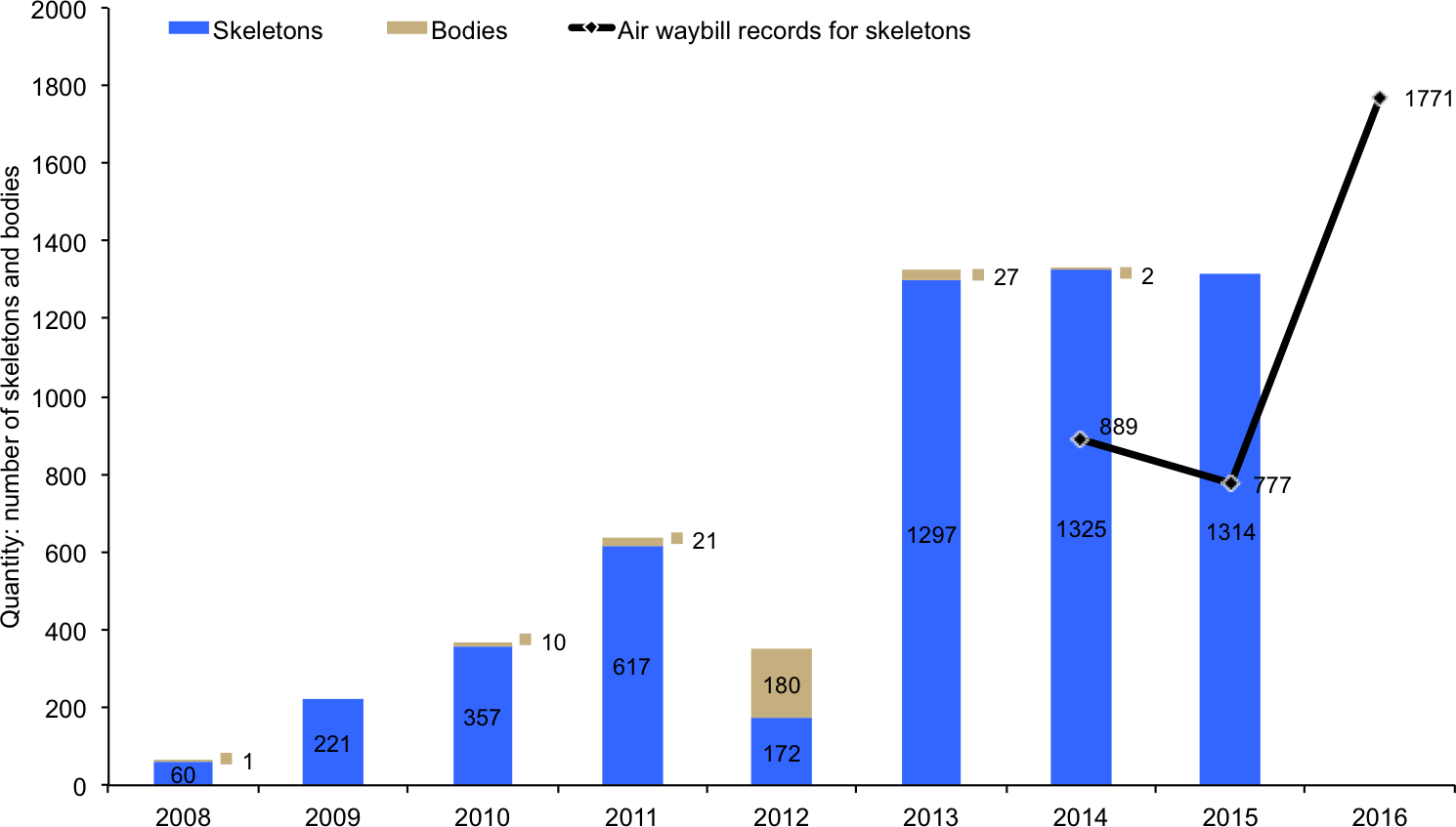
Combined number of lion skeletons and bodies sourced from Africa and listed on issued CITES permits from 2008–2015 (histogram), compared to air waybill records (black line, South Africa only) for actual exports of skeletons to East-Southeast Asia from 2014–2016. CITES permit records for skeletons and bones represent the maximum permitted annual quantity and not the actual annual exports.

Information on annual lion skeleton exports to E-SEA in 2014–2016, compiled by the freight forwarding company from the air waybills (AWB) (with the consent of their clients), revealed that 3437 sets of bones weighing 44531kg were exported to Laos, Vietnam and Thailand in three years: 889 sets in 2014; 777 sets in 2015; 1771 sets in 2016 (Fig 2). Laos received 873 sets (25%), Vietnam 2313 sets (67%), and Thailand 251 sets (7%) (Fig 3). When compared with the CITES permits issued in 2014 and 2015, the actual exports indicate that <70% of what traders applied to export in a calendar year were actually exported (see Fig 4 later).

The effect of the trade restrictions placed on Laos in 2015 is evident from the AWB data. First, total quarterly exports to E-SEA from January to September were lower than in previous years (Fig 2), resulting in an overall drop in exports for 2015 compared to 2014 (see Fig 4 later); and second, the annual exports to Vietnam and Thailand increased (Fig 3). When the trade ban was lifted, exports to Laos rose sharply in the last quarter of 2015, and stayed elevated into the first quarter of 2016 as traders resumed business and tried to catch up on lost sales (Fig 2).

The 2016 figures, however, also show a significant increase in actual exported quantities compared to previous years (Figs 2 & 4). Because of prevailing uncertainty in the industry, the surge was partly indicative of the regular availability of skeletons due to farms selling available bones monthly to South African traders rather than stockpiling them to the end of the year (which also means that bones are likely to be wetter, and the average skeleton mass heavier, than estimated by Williams et al [6]). The most evident increase was in the last quarter of 2016 following the October 2016 outcome of CoP17 that a quota on bone exports was to be implemented in 2017. The surge after CoP17 was mostly indicative of traders buying and exporting as many skeletons as possible in anticipation of a zero quota, or a quota that would be lower than the quantities that they knew could be bought from facilities [Anonymous, pers. comm., July 2017].

Another probable reason for the 2016 increase in actual exports, evident from January–September, was the U.S.’s decision to ban their hunters from importing captive origin lion trophies (notification received 19 January 2016). Since U.S. hunters usually represent ±50% of the foreign hunting clients in South Africa [2], and they imported >50% of the farmed lion trophies originating in South Africa [CITES Trade Database], it was predictable that the loss of American clients and the consequent decline in lion hunting would reduce the numbers of skeletons available for export as a by-product of the trophy hunting industry. Hence, there were legitimate concerns that breeding and hunting facilities with a surplus of lions (that were bred and/or kept for trophy hunting that could no longer be sold to foreign hunters), would reduce captive lion numbers by other means (such as euthanasia) and sell the bones–thereby increasing the potential availability of complete skeletons (i.e. with skulls) available for export (and also the average mass thereof). The quantities (sets of skeletons and mass) exported from January to September 2016 before CoP17 show a higher than average increase in exports compared to previous years, and an increase in the average mass per skeleton (see [6]). Actual exports for 2016 are more than double the quantities of previous years, and thus appear to be a reaction to the various trade restrictions that were imposed, proposed and/or anticipated. South African lion bone traders agreed that these are all valid reasons for the 2016 figures [Anonymous, pers. comm., April and July 2017]. It is further noteworthy that, while the international market for South African lion hunts has declined markedly since 2016, the domestic market has allegedly expanded (partially due to hunts being sold at reduced rates); however, South African hunters tend not to take the skulls as trophies, and so complete skeletons from trophy hunted lions are entering the supply chain more frequently [Anonymous, pers. comm., August 2017].

#### Bodies: CITES permit records

There were sporadic permit records of lion bodies exported from Africa to E-SEA prior to 2008 (average <1yr^-1^ from 2000–2007). However, quantities listed on the permits increased to ±34 bodies per year from 2008–2014 (Table 1). The total quantity for the period is 241 ‘bodies’ (98% from South Africa; reflects the adjusted data). While ‘bodies’ exported to regions besides E-SEA might typically resemble the CITES definition of ‘body’, their inclusion here is because ‘bodies’ destined for Asia were sometimes described as ‘carcasses’ on South African permit applications, and/or were confused with skeletons, and were increasingly exported after 2008. We know that trade terms have been applied inappropriately by Issuing Authorities at times and that some ‘bodies’ are actually sets of bones, possibly including some that were not thoroughly cleaned and prepared and which are therefore heavier than boiled and/or taxidermied specimens. A trader believes that most, if not all, “*bodies on the permits are actually skeletons incorrectly captured*” [Anonymous, pers. comm., July 2017]. The South African provincial exporters of lion bodies annually to E-SEA are in S1 Table.

#### Combined exports of skeletons and bodies

The combined maximum permitted legal export of lion skeletons and bodies from Africa to E-SEA from 2008–2015 (based on permits issued) amounts to 5604 units (SKE & BOD), 50% of which were destined for Laos and 45% to Vietnam (Table 1). The quantities average 322yr^-1^ from 2008–2011, but 1322yr^-1^ from 2013–2015 (Fig 4). The only African countries reporting legal exports of these products are South Africa, Namibia, Tanzania and Zimbabwe.

The 2014–2015 AWB records are the only confirmations we have of actual exports relative to the permits that were issued by Parties (Fig 4). However, these annual figures must be viewed in the context of interpreting the limitations of the CITES reports, namely that: (i) actual exports do not necessarily occur in the same year that the CITES permit was issued (especially if the permit was issued towards the end of a calendar year, in which case skeletons will be exported at the beginning of the following year), and (ii) the fidelity of the data depends on the accuracy and completeness of the information submitted to the Secretariat (including whether Parties submit their reports for permits that were issued in a calendar year). The 2016 exports confirmed by the South African AWB data (black line in Fig 4) partially show the trajectory of actual trade had the quota not been implemented, albeit with the additional factors in evidence that relate to the various market uncertainties that probably resulted in more annual exports than might have otherwise occurred.

#### Wild-sourced or captive-produced?

Most lion skeletons and bodies originating from South Africa are listed on permits as captive-bred (C) (94% and 69% respectively) (Table 3). In addition, five bodies and 47 skeletons (all wild-sourced) were listed on permits from other African countries. However, the actual proportion of wild-sourced bodies and skeletons from South Africa is less than these data show because some provincial permit Issuing Authorities were, until early 2012, erroneously recording some captive-produced lions as ‘wild sourced’. This error happened because certain Issuing Authorities misinterpreted the regulation that lions must be ‘free-roaming’ for a specified period before they can be hunted. Some took this to mean that captive-bred lions could be considered ‘wild-origin’ if they were hunted after the mandatory release period set by the province. However, this free-roaming release period before trophy hunters can hunt ranges from four days to 24 months, depending on the South African province (see page 23 Williams et al [2]), and captive-bred lions can never be reclassified as wild. Hence the proportion of ‘wild-sourced’ lion over the period 2008–2015 is erroneously elevated to an unknown degree and is likely to be closer to zero.

**Table 3:** Number and proportion of wild-sourced and captive-bred lion bodies and skeletons originating in South Africa listed on issued CITES permits and destined for East-Southeast Asia from 2008–2015.

	Wild-sourced (W) ^a^^,^^b^	Captive-bred (C)	Other ^c^	Total
**Skeletons**	315 (6%)	4981 (94%)	20 (0.4%)	5316
**Bodies**	71 (30%)	164 (69%)	1 (0.4%)	236

^a^ Figures for wild-sourced lion skeletons and bodies from South Africa are elevated to an unknown degree due to a misinterpretation prior to 2012 of the meaning of ‘wild’

^b^ Excluded from the wild-sourced column are 47 skeletons from Namibia and 5 bodies (from Namibia, Tanzania or Zimbabwe)

^c^ 20 Unknown (U); 1 Ranched (R)

#### Purpose of trade

**Fig 5:**
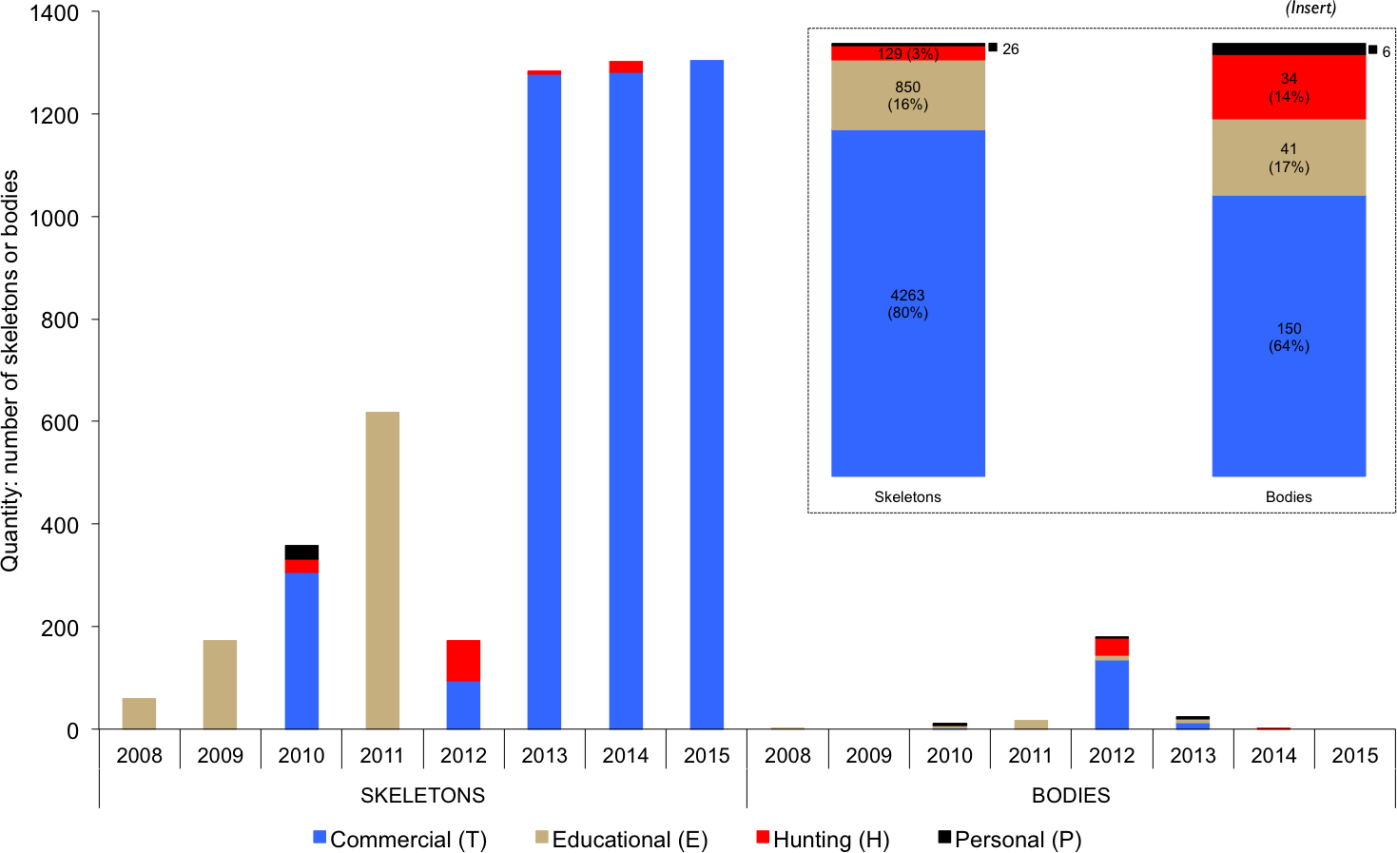
Annual number of lion skeletons and bodies by purpose code originating in South Africa listed on issued CITES permits and destined for East-Southeast Asia from 2008–2015. In addition, permits for five bodies and 47 skeletons, all for commercial purposes, were issued from other African countries in the same period. The insert shows the total quantity and percentage for skeletons and bodies. See S1 Fig for the purpose codes for skeletons on permits issued to Laos, Vietnam, Thailand and China.

The purpose of trade listed for most lion skeletons and bodies originating in South Africa from 2008–2015 was commercial (T) (80% and 64% respectively) (Fig 5 *insert*). However, ‘educational’ was listed as the sole purpose for skeletons in 2008, 2009 and 2011 (amounting to 850 SKE) (Fig 5), and nearly 30% of the quantity to Laos was for this code (S1 Fig). From 2013, however, the purpose of nearly all skeleton exports was captured as commercial on issued permits (Fig 5).

A clue to why the early exports of lion bones were listed as being for ‘educational’ purposes might lie in the investigation of Asia’s animal trafficking network by Davies & Holmes [54]. Companies operating wildlife farms in Laos are alleged to have imported captive-bred tigers, *“which was legal as long as they were used for science and education*, *not for commercial trade”* [54]. The Keosavang Trading Company, a Laos-based importer of South African bones, was one of the companies named to be operating a wildlife farm in Laos [54]. Thus, in order to export lion bones legally from South Africa, did traders initially copy the established procedures for purchasing tigers in Asia by declaring consignments ‘educational’?

## Overview

It is evident that the trade in lion bones is a complex issue that spans continents and cultures with a mosaic of stakeholders. From the results of both our investigations (this one and [8]), there appear to be at least three supply-related trade chains in the market for lion bones across Asia and Africa that stimulate and support the demand. First, the legal but rapacious E-SEA trade, derived annually from hundreds of captive-bred South African lions and a limited number of wild-origin ones from other African countries (the latter amounting to 0.9% of the total quantity listed on issued CITES permits to 2015, all from Namibia, Table 1). Second, the same trade to E-SEA, but conducted illegally (e.g. no permits, or sourced by poaching) (not quantified here). And third, the relatively more widespread but comparatively modest, but no less threatening, pan-African utilisation of bones that is mostly for ‘traditional’ purposes and is typically sourced outside South Africa from wild lion populations killed in human-lion conflicts (‘problem lions’) and poaching [8], or in South Africa from poaching of captive-bred lions or sales from captive-facilities to traditional medicine (‘*muti*’) traders [Anonymous, pers. comm., August 2017]. A South African trader was hesitant when asked about whether more frequent media reports of incidences of lion poaching in South Africa since c.2015 were signs that there has been an actual rise in poaching attributable to the Asian trade compared to the *muti* trade [Anonymous, pers. comm., August 2017]–hence, substantiated evidence for this is required.

The domestic trade in lion bone is, along with other cultural and socio-economic drivers of lion utilisation, and to different degrees, an anthropogenic threat to wild lion populations across the African continent. The transnational market network is typically supplied through captive-bred and privately-owned lions in South Africa (mostly trophy hunted), or wild lions procured illegally by wildlife harvesters in other countries (to an unknown extent). Since the Asia-driven tiger parts trade provoked and aggravated negative consequences for lions and other large felids, pertinent questions are being asked about how the lion bone trade counter-influences the tiger trade. Furthermore, (i) to what extent is lion poaching that is directly attributable to the Asian and broader pan-African domestic trades occurring in South Africa and other African countries; (ii) what are suppliers and importers doing with the lion bones, (iii) are bones being processed into products prior to export to evade detection and circumvent the mandatory permit regulations (and hence to what extent), and (iv) are lion bones that are processed and sold to Asian consumers being marketed as lion or tiger? Accordingly, these questions merit further scrutiny, as the body of evidence is limited and/or has not been accessed yet.

Regarding the legal global wildlife trade, the CITES Trade Database is the best available source of information; however, there are limitations to using it as a proxy for assessing the actual amount of illegal trade [74] including, that not all illegal transactions are detected and seized, and not all seizures are reported to the Secretariat by Parties. Relatedly, the database (i) is not a suitable proxy for estimating the total number of skeletons in the resource base derived from trophy hunting (for this, one needs to examine national hunting registers [2]), and (ii) is an imprecise proxy for quantifying the total number of skeletons of individual lions entering into the bone trade. CITES trade data obtained from the UNEP-WCMC database have several inherent deficiencies that must be noted in any discussion on their usefulness and accuracy. For example, most CITES permits are issued to traders based on the number of specimens stated in their application documents. As relatively few governments check the actual number of specimens exported, it is usually hard to say whether exports are higher or lower than stated on the permit unless the permits are endorsed. Furthermore, although importing and exporting countries are meant to record the volume of shipments at both ends, this often does not occur and so once again it may be impossible to confirm the volume of actual imports. Confounding CITES data, which generally represents legal trade, is the fact that in some high value species there is often an undocumented parallel illegal trade–the size of which has yet to be assessed for lions. Therefore, to improve confidence in CITES trade data it is necessary to corroborate it by comparison against independent data obtained from field surveys of legal suppliers and/or illegal harvester activities and/or illegal trade in consumer nations–which is what we were partly able to do through access to the AWB records for legal bone exports. The more independent data sets that can be used to check CITES permit data, the better it is for deciding its reliability.

From 2017, South Africa is the only country legally authorised to export lion skeletons to E-SEA–but attempts to illegally procure and trade in lion body parts and bones (from wild and captive lions) will persist (most likely in neighbouring countries [Anonymous, pers. comm. August 2017]), and thus requires vigilance and monitoring. Illegal trade includes attempts to smuggle parts (e.g. teeth and claws, for which there is no quota) and bones to E-SEA and elsewhere that (i) exceeds the allocated quota and does not comply with permit regulations, (ii), originates from other countries, or (iii) are wild-sourced. However, depending on the range state, intercepted illegal consignments might not necessarily originate from wild lions. In South Africa, provided a ToPS (Threatened or Protected Species) permit has been issued permitting restricted activities for lions (e.g. possessing, buying, moving, receiving, etc), the domestic trade in lion parts is generally legal. Therefore, it is not inconceivable that captive facilities might sell lion parts to persons with ToPS permits who ultimately intend exporting them illegally (as a whole, in parts, or processed).

## Conclusion

The trade in lion bones from South Africa to E-SEA has risen consistently since 2008, as evidenced by the quantities recorded on the issued CITES permits. If actual exports were at least ±89% of the permitted quantity, then bones from ±2621 individual lions were exported from 2008–2013. In addition, AWB records show that a further 3437 skeletons were actually exported from 2014–2016, bringing the estimated total from Africa to E-SEA in the period 2008–2016 to around 6058 skeletons (i.e. no less than 70 metric tonnes, 64% in the last three years from 2014). While the CoP17 annotation restricting trade to captive-origin bones from South Africa only will change the trajectory of legally exported bone quantities, of concern is the trajectory and *modus operandi* for illegal trade since incidences of poaching are recurrently reported across the African continent; these incidences in South Africa, however, mostly end in the removal of teeth, paws and claws from privately-owned lions, seemingly to supply separate markets for these products in Africa and Asia [8; Anonymous, pers. comm., August 2017].

The international trade in lion bones to E-SEA for tonics/medicines coexists with the more widespread trade in lion bones and body parts for mainly zootherapeutic purposes across the African continent [see 8]. In African lion range states with no farmed lions, and/or those with smaller and/or less protected wild populations, vulnerability to poaching is informed by the drivers of trade and the magnitude thereof. While there is minimal evidence to suggest that the East-Southeast Asian bone trade is presently adversely affecting wild lions in protected areas in South Africa, the extent of this specific trade in other lion range states still requires urgent proactive monitoring and evaluation to substantiate and clarify these impacts and also those resulting from the trade in lion body parts for other purposes. And, of particular concern are reports of Asian nationals enquiring about lion bones in Eastern and Southern African lion range states [8], and the evidence of at least one consignment exported from Uganda to Laos in 2016, because this implies deliberate bioprospecting and a more organised and less opportunistic approach to sourcing and acquiring wild lion body parts and bones.

## Supporting information

S1 Table South African provincial exporters of lion bodies to East-Southeast Asia from 2008–2015. (PDF)

S1 Fig Purpose codes on CITES permits issued for skeletons destined for Laos, Vietnam, Thailand and China. (TIFF)
