# Supplementary material for: A roaring trade? The legal trade in Panthera leo bones from Africa to East-Southeast Asia
Source: PLoS One. 2017 Oct 24;12(10):e0185996. doi: 10.1371/journal.pone.0185996 (PMC5655489; doi:10.1371/journal.pone.0185996)
Supplement: S1 Table — (PDF) [file pone.0185996.s001.pdf]

S1 Table. South African provincial exporters of lion bodies to East-Southeast Asia from 2008–2015.

Year	Free State	North West	Mpumalanga	Unknown	*Total*
2008				1	*1*
2009					
2010		2	6*	2	*10*
2011			16	5	*21*
2012	14		12	153	*179*
2013				23	*23*
2014				2	*2*
2015*					
Total no. BOD	*14*	*2*	*34*	*186*	*236*
Total %	*6%*	*1%*	*14%*	*79%*	

* Two white lion cubs, and four bodies for museum display only
