# Supplementary figures and images for: A roaring trade? The legal trade in Panthera leo bones from Africa to East-Southeast Asia
Source: PLoS One. 2017 Oct 24;12(10):e0185996. doi: 10.1371/journal.pone.0185996 (PMC5655489; doi:10.1371/journal.pone.0185996)

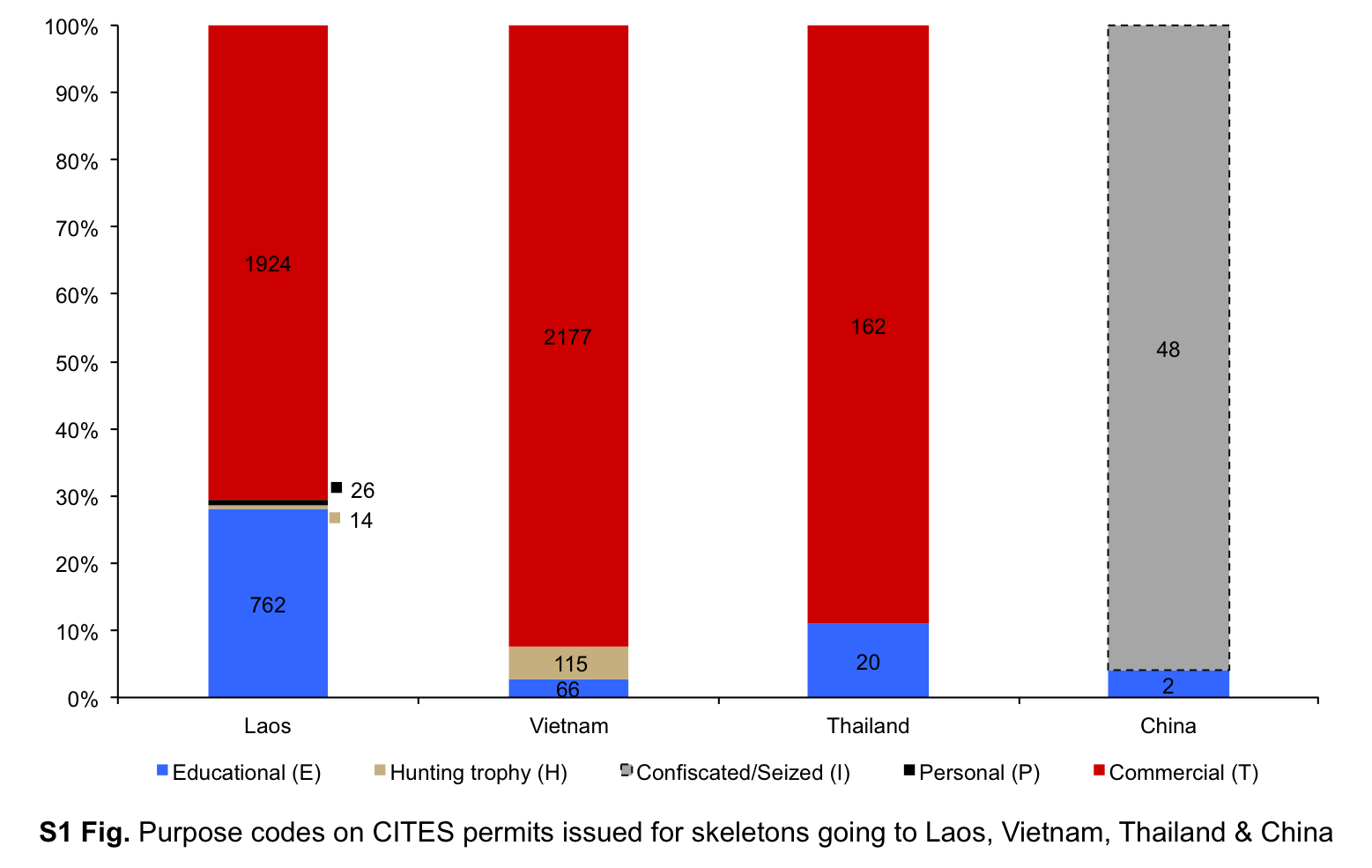

Supplement: S1 Fig — (TIFF) [file pone.0185996.s002.tiff]
